# Keystone Veterinary Medical Association

**Published:** 1895-05

**Authors:** W. L. Rhoads

**Affiliations:** Secretary


Secretary.

### KEYSTONE VETERINARY MEDICAL ASSOCIATION.

The regular monthly meeting of the Keystone Veterinary Medical Association was held at the office of Dr. W. S. Kooker, 662 N. Eleventh St., Phila., on Tuesday evening, April 9th. The meeting was called to order by the election of Dr. H. P. Eves President *pro tem*, after which the Secretary called the roll.

The report of the Legislative Committee conveyed to the members the standing of the present proposed legislation at Harrisburg, and urged the members to use their influence with the Senators to forward the Live Stock Sanitary Board Bill, and also the bill establishing a State Board of Veterinary Medical Examiners which had that day passed the House on third reading by a vote of 147 to 11.

The resignations of Dr. A. T. Sellers and W. L. Nunan were on recommendation of the Board of Trustees accepted by the Association.

A number of interesting cases were reported and discussed.

It was decided to hold the June meeting, which would be the last of the year, at Lansdowne, on invitation of Secretary Rhoads.

W. L. Rhoads,
Secretary.

